# Exosomal non-coding RNAs: a new avenue for treating diabetic foot ulcers

**DOI:** 10.3389/fmolb.2025.1701879

**Published:** 2025-12-08

**Authors:** Guohao Chen, Gang Chen, Jun Lu, Shaolong Hu

**Affiliations:** 1 Department of Traumatology, Jinhua Municipal Central Hospital, Jinhua, Zhejiang, China; 2 Department of Interventional Medicine, Jinhua Municipal Central Hospital, Jinhua, Zhejiang, China; 3 Department of Laboratory Medicine, Jinhua Municipal Central Hospital, Jinhua, Zhejiang, China

**Keywords:** diabetic foot ulcer, non-coding RNA, exosome, wound healing, therapeutic strategy

## Abstract

Diabetic foot ulcer (DFU) is a severe complication resulting from diabetes mellitus (DM) that affects approximately 18.6 million individuals annually and has a lifetime incidence of up to 25% among DM patients. These ulcers often precede lower-extremity amputations and are associated with high mortality as well as economic burden that necessitate innovative therapeutic strategies beyond conventional methods. Recent research efforts have highlighted the potential of non-coding RNAs (ncRNAs), including microRNAs (miRNAs), long non-coding RNAs (lncRNAs), and circular RNAs (circRNAs), that regulate gene expression and cellular functions critical for wound healing. Exosomes are the natural carriers of ncRNAs and offer a promising avenue for the treatment of DFU by enhancing the stabilities and bioavailabilities of these molecules. In this review, we explore the substantial potential of ncRNAs in DFU treatment by emphasizing the action mechanisms of ncRNAs, refinement of exosome-based delivery systems, and expansion of clinical trials to translate ncRNA-based therapies into clinical practice. The application of exosomal ncRNAs involves diverse strategies through different mechanisms, although there remain challenges in terms of exosome preparation consistency, functional enhancement, and efficient drug delivery. The future directions in this regard include optimizing isolation techniques, engineering exosomes for improved targeting, integrating with biomaterials, and conducting more clinical trials to validate safety and effectiveness, thereby paving the path for widespread clinical use.

## 1 Introduction

Diabetic foot ulcers (DFUs) represent a significant complication of diabetes mellitus (DM) and pose serious threats to the health and quality of life of the patients. Globally, approximately 18.6 million individuals with DM are affected by DFUs annually, with a lifetime incidence of up to 25% among DM patients (Armstrong et al., 2023). These ulcers often precede lower-extremity amputations, and approximately 80% of such amputations are linked to DFUs. The presence of DFU not only increases the risk of amputation but also elevates the mortality rate. Patients with DFUs have a 5-year mortality rate of approximately 30%, which can increase to over 70% for individuals who have had major amputations (Lim et al., 2017); the economic burden is also substantial, with DFU-related complications accounting for a considerable proportion of the healthcare costs borne by DM patients. In the United States alone, the annual direct medical costs associated with the treatment of DFUs are estimated to be of the order of billions of dollars (Armstrong et al., 2020). The high prevalence and severe consequences of DFUs highlight the importance of early detection, prevention, and effective management (Rehman et al., 2023). Multidisciplinary approaches involving the integrated efforts of healthcare professionals from various specialties have been shown to reduce amputation rates and improve patient outcomes.

Dysregulated lipogenesis and excessive lipid accumulation are some of the pivotal upstream drivers of DM and subsequent risk of DFUs (Yang et al., 2024). Lipogenesis is the *de novo* synthesis of fatty acids from carbohydrates that is primarily regulated by the transcription factors PPARγ and SREBP-1c and occurs predominantly in the liver, adipose tissue, and mammary glands (Jeon et al., 2023). Under physiological conditions, lipogenesis supports energy storage; however, chronic overnutrition (e.g., high-sugar/high-fat diets) can induce hyperactive lipogenesis and lead to abnormal lipid accumulation (Sanders and Griffin, 2016). Ectopic lipid deposition (e.g., in the skeletal muscles, pancreas, and vascular walls) can trigger two critical events. The first is pancreatic β-cell dysfunction, where lipotoxicity impairs insulin secretion by disrupting mitochondrial functions and increasing the stress on the endoplasmic reticulum (Prentki and Madiraju, 2012). The second is peripheral insulin resistance, in which free fatty acids (FFAs) and lipid metabolites (e.g., ceramides) inhibit insulin signaling in the adipocytes, myocytes, and hepatocytes to reduce glucose uptake and utilization (Prentki and Madiraju, 2012). Together, these events promote the development of type 2 DM. Sustained lipid metabolic disorders in diabetic patients can further exacerbate the risk of DFUs; vascular endothelial cells damaged by lipotoxicity exhibit reduced angiogenesis (via suppressed VEGF-A expression) (Prentki and Madiraju, 2012), while peripheral neurons undergo lipid-induced oxidative stress and inflammation, leading to sensory/motor neuropathy (Singleton et al., 2022). These impairments compromise wound perfusion, tissue repair, and infection clearance, which are the key factors contributing to DFU initiation and non-healing (Wukich et al., 2017).

Despite the availability of various therapeutic modalities for DFUs, the healing process remains a challenge. Conventional treatments for DFUs include offloading, wound debridement, infection control, and vascular reconstruction (Senneville et al., 2024). Offloading techniques, such as total contact casts and therapeutic shoes, aim to reduce the pressure on the affected area to promote healing (Bellomo et al., 2022). Wound debridement involves the removal of necrotic tissues to enhance the wound environment (van Netten et al., 2024). Infection control is crucial and often requires antibiotics as well as surgical interventions in severe cases. Vascular reconstruction entails procedures necessary to improve blood flow to the affected limb (Castellin et al., 2025). Despite these efforts, the high prevalence and severe consequences of DFUs highlight the need for innovative therapeutic strategies beyond conventional methods.

There is an accumulating body of evidence underscoring the pivotal roles of non-coding RNAs (ncRNAs), including microRNAs (miRNAs), long non-coding RNAs (lncRNAs), circular RNAs (circRNAs), and other ncRNAs, in orchestrating gene expression and cellular functions, thereby presenting new opportunities for DFU management (Kowluru and Mohammad, 2022). These molecules exert fine control over some essential biological processes like inflammation, angiogenesis, re-epithelialization, and extracellular matrix remodeling, all of which are indispensable for effective wound repair and regeneration (Mohsin et al., 2024). For example, during the inflammatory phase, miR-146a attenuates inflammatory responses by inhibiting the NF-κB pathway (Poe et al., 2022), while miR-132 facilitates the transition from inflammation to proliferation (Essandoh et al., 2016). Additionally, miR-21 concurrently reduces inflammation while promoting cellular proliferation and migration (Hu et al., 2018). The lncRNA H19 has been reported to enhance wound repair by targeting miR-152-3p, and circRNAs can modulate cell functions through interactions with miRNAs or proteins (Li et al., 2020).

Exosomes have garnered significant attention as natural carriers of ncRNAs (Pegtel and Gould, 2019), offering a promising avenue for augmenting DFU treatment. These extracellular vesicles can effectively deliver ncRNAs to the target cells, thereby enhancing their stability and bioavailability. Exosomes derived from mesenchymal stem cells (MSCs) and adipose-derived stem cells (ADSCs) have demonstrated potential in carrying specific miRNAs, lncRNAs, and circRNAs that modulate intracellular signaling pathways and gene expression, significantly promoting DFU healing (Gao et al., 2018).

The present review explores the substantial potential of ncRNAs in the treatment of DFUs by emphasizing the action mechanisms of ncRNAs, refinement of exosome-based delivery systems, and expansion of clinical trials to translate ncRNA-based therapies into clinical practice.

## 2 Different types of ncRNAs and their biological functions

**FIGURE 1:**
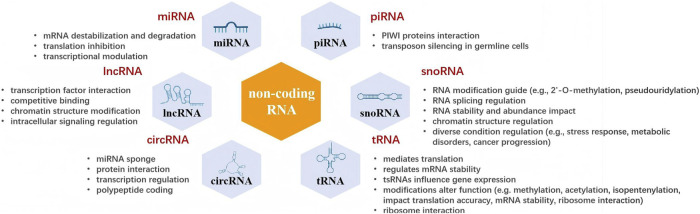
Types and molecular functions of exosomal non-coding RNAs. miRNAs, piRNAs, lncRNAs, snoRNAs, circRNAs and tRNAs are sorted into functional modules (coloured hexagons) that illustrate their principal modes of action: mRNA destabilisation, translational control, epigenetic modulation, chromatin remodelling, miRNA sponging, RNA modification guidance and ribosome interaction. The central “non-coding RNA” hexagon emphasises that these ncRNA classes collectively fine-tune gene expression and cellular homeostasis.

In recent years, ncRNAs have emerged as crucial regulators of the pathogenesis and development of DM and its complications, including DFUs (Gao et al., 2023). These ncRNAs, including miRNAs, lncRNAs, circRNAs, piwi-interacting RNAs (piRNAs), small nucleolar RNAs (snoRNAs), and transfer RNAs (tRNAs), are known to participate in diverse biological processes through various mechanisms like gene expression regulation, epigenetic modification, and post-transcriptional processing (Al-Haddad et al., 2016). In this section, we focus on the current understanding regarding the roles of ncRNAs in metabolic diseases and offer insights into their regulatory mechanisms (see Figure 1).

### 2.1 MicroRNA

miRNAs are small endogenous single-stranded RNA molecules of typically 20–23 nucleotides in length that were first identified in 1993 (Saliminejad et al., 2019; Lee et al., 1993). They primarily function by binding to the 3′ untranslated region (3′-UTR) of the target messenger RNA (mRNA), leading to negative regulation of gene expression through mRNA degradation or translation inhibition (Shang et al., 2023). Second, miRNAs can inhibit protein synthesis by preventing the binding of translation factors to mRNAs through interactions with 3′-UTR, resulting in decreased production of the target gene proteins. This translation inhibition is one of the principal pathways by which miRNAs regulate gene expression (Kara et al., 2022). Additionally, certain miRNAs can modulate transcriptional activities by interacting with chromatin regulatory factors, thereby allowing control of gene expression at earlier stages (Kara et al., 2022; Zan et al., 2014). However, it should be noted that miRNAs can also bind to the 5′ untranslated region (5′-UTR) or even the entire mRNA, although these occurrences are less common (Lee et al., 2009). When miRNAs bind to the 5′-UTR, they can enhance translation by stabilizing the mRNAs or facilitating the binding of translation initiation factors (Strayer et al., 2024). For instance, miR-10a binds to the 5′-UTR of ribosomal protein mRNAs and enhances their translation by stabilizing the mRNAs (Ørom et al., 2008). Similarly, miR-346 targets the 5′-UTR of the RIP140 mRNA, leading to increased protein expression (Tsai et al., 2009). In some cases, the miRNA can bind to the entire mRNA to influence its overall stability and translation efficiency. For example, miR-483-5p binds to the 5′-UTR of the IGF2 mRNA to promote transcription and translation (Chen et al., 2025).

These diverse regulatory mechanisms enable miRNAs to play crucial roles in various cellular processes, such as proliferation, apoptosis, differentiation, and migration (Ambros, 2003). Consequently, miRNAs have significant impacts on the development and progression of metabolic disorders, including DM. By understanding these mechanisms, we can better appreciate the potential therapeutic implications of miRNAs in metabolic diseases.

### 2.2 Long non-coding RNA

lncRNAs are a class of ncRNAs exceeding 200 nucleotides in length that are primarily transcribed by RNA polymerase II and generally lacking in terms of protein-coding capacity (Djebali et al., 2012). While some lncRNAs may share partial structural features with mRNAs, they fundamentally differ from mRNAs in both genomic origin and molecular characteristics. In terms of the genomic encoding regions, lncRNAs are predominantly transcribed from the intergenic regions, introns of protein-coding genes, or antisense strands of the coding loci, whereas mRNAs are specifically derived from the exonic regions of protein-coding genes (Mattick et al., 2023; Zhang, 2024; Poliseno et al., 2024). In terms of the molecular features, lncRNAs typically have fewer exons, have lower sequence conservation across species, and often exhibit tissue-specific or cell-type-specific expression patterns (Zhang et al., 2022; Ahmadi et al., 2025). In contrast, mRNAs have more structured exonic arrangements, greater sequence conservation, and broader expression profiles in functionally related cells (He et al., 2023a; Ginzburg et al., 1982). Notably, unlike mRNAs, most lncRNAs lack a canonical open reading frame (ORF) and cannot be translated into proteins, which further distinguish them from mRNAs (Xiao et al., 2024). This characteristic, coupled with their typically low expression levels and species-specific nature, led to their early mischaracterization as non-functional transcriptional noise (Graf and Kretz, 2020). However, their presence in both the nuclear and cytoplasmic compartments suggests significant roles in diverse biological processes, including gene transcription, post-transcriptional modifications, genome packaging, maintenance of cellular structural integrity, chromatin remodeling, and modulation of protein intracellular localization (Michieletto and Henao-Mejia, 2021).

Functionally, lncRNAs can be classified into four main categories (Tan et al., 2021). First, some lncRNAs can regulate target gene transcription through interactions with specific transcription factors or regulatory proteins (Blythe et al., 2016). Second, certain lncRNAs can modulate gene expression at the transcriptional and post-transcriptional levels by competitively binding with other ncRNAs like miRNAs (Wang et al., 2017). Third, some lncRNAs are known to influence gene expression by modifying the chromatin structures or affecting the post-transcriptional processes (Martens et al., 2021). Fourth, certain lncRNAs can shuttle between the nucleus and cytoplasm, where they are processed into other ncRNAs to regulate the intracellular signaling pathways and metabolic processes (Jathar et al., 2017).

As key regulators of metabolic tissue development and functions (Kornfeld and Brüning, 2014), altered lncRNA expression levels can disrupt metabolic homeostasis and contribute to disease onset (López-Noriega and Rutter, 2020). Genome-wide association studies have identified some of the lncRNAs linked to the diabetes susceptibility loci (Hanson et al., 2007), implying the significant regulatory roles of lncRNAs in diabetes progression. This evidence underscores the potential of lncRNAs as therapeutic targets in metabolic diseases.

### 2.3 Circular RNA

circRNAs are characterized by their covalently closed circular structures and have emerged as a significant class of ncRNAs (Yang et al., 2021). They were initially regarded as rare byproducts of aberrant RNA splicing, a notion that was first proposed in early studies on plant viroids and later observed in mammalian cells (Sanger et al., 1976; Capel et al., 1993), but have been increasingly identified and recognized as functional molecules in mammalian cells with the advent of RNA deep sequencing and bioinformatics tools (Capel et al., 1993; Memczak et al., 2013). Numerous studies have demonstrated that circRNAs can function as miRNA sponges to regulate gene expression at the post-transcriptional level (Kristensen et al., 2019). This discovery has spurred extensive research into the biological properties, functional mechanisms, and potential clinical applications of circRNAs in diagnostics and therapeutics.

As one of the most distinctive features of circRNAs, the circular structure confers high resistance to exonucleolytic degradation and enhances stability compared to linear RNAs (He et al., 2021). The absence of the 5′ cap and 3′ poly-A tail structures results in predominant cytoplasmic localization, although certain types like the exon–intron circRNAs and intron-derived circRNAs are localized in the nucleus (Zhou et al., 2020). This stability allows circRNAs to accumulate within the cells and exert their biological functions over extended periods of time (Pervouchine, 2019).

circRNA biogenesis primarily involves alternative splicing of the precursor mRNA (pre-mRNA) through a process termed as back-splicing. In back-splicing, a downstream splice acceptor site is joined to an upstream splice donor site to form a circRNA molecule (Di Timoteo et al., 2020). The mechanisms underlying back-splicing include intron-pairing-driven circularization, RNA-binding protein-mediated circularization, and lariat-driven circularization resulting from exon skipping (Wawrzyniak et al., 2020). These intricate processes are regulated by multiple cis-acting elements and trans-acting splicing factors (Zhao et al., 2022).

Several studies have demonstrated that circRNAs play crucial roles in cellular physiological processes. circRNAs contain numerous miRNA binding sites and can function as competitive endogenous RNAs by binding to miRNAs, thereby preventing their inhibitory effects on the target mRNAs and indirectly regulating gene expression (Qadir et al., 2022). For instance, circHIPK3 promotes cellular proliferation by sequestering multiple distinct miRNAs (Zhang et al., 2020). Moreover, circRNAs can interact with proteins, acting as protein scaffolds or competitively binding proteins to influence protein functions (Ma et al., 2025). For instance, circFoxo3 interacts with CDK2 and p21 to form a ternary complex, inhibiting CDK2 function and affecting the cell cycle (He et al., 2023b). circRNAs can also regulate transcription; some circRNAs can bind to RNA polymerase II to enhance or suppress transcription of the parental genes. circEIF3J and circPAIP2 are two exon-intron circRNAs that interact with RNA polymerase II, U1 small nuclear ribonucleoprotein particles (snoRNPs), and host gene promoters to boost host gene transcription (Lu and Xu, 2016). Although most circRNAs are non-coding in nature, some can translate into polypeptides under specific conditions to exert regulatory functions. Under stress conditions, certain circRNAs can also serve as internal ribosome entry sites to direct polypeptide synthesis (Fan et al., 2022).

The stability and functional diversity of circRNAs make them highly promising molecules for applications in disease diagnosis and treatment, particularly in complex conditions like DFUs, where circRNAs could serve as novel therapeutic targets and biomarkers. In the future, studies are expected to further explore the specific roles of circRNAs in various diseases to provide a theoretical basis for the development of new circRNA-based therapeutic strategies.

### 2.4 Other non-coding RNAs

Other ncRNAs include piRNAs, snoRNAs, and tRNAs. piRNA is an ncRNA that is approximately 21–35 bp in length and is primarily isolated from the germ cells of mammals (Ma et al., 2025). It forms a complex with the members of the PIWI protein family to silence target genes for regulatory purposes (Stallmeyer et al., 2024). Many studies have indicated that piRNAs can modulate cellular physiological and pathological processes by regulating cellular proliferation, apoptosis, and angiogenesis (Zhang et al., 2023a). For instance, piRNA-48383 has been linked to insulin resistance via participation in the circulation of extracellular RNAs and epigenetic modifications (Wang et al., 2023a). Moreover, the overexpression of DQ732700 and DQ746748 piRNAs in the pancreatic islet cells of diabetic rats has been shown to suppress glucose-induced insulin release (Chu et al., 2015).

SnoRNAs are small ncRNAs of length 60–300 bp that are found in the nucleolus of eukaryotic cells and can bind to snoRNP complexes (Liu et al., 2025). In vertebrates, the snoRNA genes are mainly located in the introns of the protein-coding or non-protein-coding genes and undergo further post-transcriptional processing to form mature snoRNAs (Monzian et al., 2023). SnoRNAs regulate gene expression through multiple mechanisms. First, snoRNAs guide RNA modifications, including 2′-O-methylation and pseudouridylation of ribosomal RNA (rRNA), tRNA, and small nuclear RNA (snRNA), which are crucial for the ribosome structure and protein synthesis efficiency (Chauhan et al., 2024). Second, snoRNAs regulate RNA splicing; some of them bind to pre-mRNAs to influence alternative splicing, increase gene expression diversity, and affect the splicing of other RNAs, thereby modulating their functions and stabilities (Wa et al., 2021). Third, snoRNAs can impact RNA stability and abundance; some are processed into miRNAs that can bind to target mRNAs to inhibit translation or induce degradation, thereby regulating mRNA stability and abundance directly (Xing and Chen, 2018). Lastly, snoRNAs regulate the chromatin structure by interacting with chromatin to influence its condensation and accessibility to control gene transcription activity (Nahkuri and Paro, 2012). Consequently, snoRNAs play significant roles in gene expression regulation through diverse mechanisms by impacting cellular processes and disease development.

Recent data suggest that snoRNAs may be involved in the regulation of various conditions, including genetic diseases, hematopoiesis, metabolism, and cancer (Xiao et al., 2023). To investigate the role of the Rpl13a snoRNA in systemic glucose metabolism, Lee et al. (2016) developed Rpl13a-snoRNA-deficient mice; subsequent glucose tolerance tests revealed that the Rpl13a-snoRNA-knockout mice exhibited significantly enhanced glucose tolerances and higher serum insulin levels than wild-type mice, indicating the potential of the Rpl13a snoRNA as a biomarker for diabetes.

The tRNAs are molecules typically consisting of 70–90 nucleotides that can mediate the pairing of amino acids with their corresponding codons on mRNAs (Chen et al., 2021). In recent years, tRNAs have been found to be involved in not only protein synthesis but also gene expression regulation, cell cycle control, and stress responses (Wang et al., 2023b). During translation, tRNAs play crucial roles in transporting amino acids to the ribosomes for protein synthesis according to the genetic codes on the mRNAs (Zhou et al., 2019). Moreover, tRNAs can regulate mRNA degradation; for instance, the tRNA specific to decoding arginine codons can recruit the CCR4–NOT complex to the translating ribosome to trigger mRNA degradation and turnover, which is a mechanism vital for mRNA stability in mammalian cells and particularly for mitochondria-related mRNAs (Buschauer et al., 2020).

tRNA-derived small RNAs (tsRNAs) are also known to significantly impact gene regulation; they can interact with transcription factors or other regulatory proteins to influence transcription initiation and processes, process transcription products to affect their stabilities and functions, and interact with ribosomes or translation initiation factors to affect protein synthesis rates and quality (Zhang et al., 2023b). tRNA modifications can also influence functions and gene expression. These modifications affect the tRNA’s structural stability, amino acid binding ability, and interactions with ribosomes to impact protein synthesis efficiency and accuracy (Akiyama and Ivanov, 2023). Abnormal tRNA modifications are closely linked to dysregulated lipogenesis, lipid accumulation, and subsequent risk of diabetes/DFUs. For instance, tRFGluTTC accumulates in the perirenal adipose tissue and inhibits preadipocyte differentiation by downregulating the lipogenesis-related genes (Shen et al., 2019). This inhibition disrupts normal lipogenesis; while moderate lipogenesis supports adipose tissue expansion to store excess energy, tRFGluTTC-mediated suppression shifts the lipid deposition toward ectopic sites rather than subcutaneous adipose tissues. Ectopic lipid accumulation triggers lipotoxicity, which is characterized by the release of FFAs and proinflammatory cytokines that impair insulin signaling by phosphorylating IRS-1 at serine residues; this leads to insulin resistance, which is a primary driver of type 2 DM (Shen et al., 2019; Shen et al., 2018; Peng et al., 2012). In diabetic patients, sustained insulin resistance has been known to exacerbate vascular endothelial dysfunction and peripheral neuropathy, weakening tissue perfusion and the wound repair capacity (Fang et al., 2024; Clyne, 2021). These changes, coupled with impaired immune responses to infection, can significantly increase the risk of developing DFUs (Yang et al., 2024). Thus, tRFGluTTC-mediated dysregulation of lipogenesis and lipid accumulation represents a key molecular link between tRNA dysfunction, diabetes, and susceptibility to DFUs.

The interactions between tRNAs and ribosomes are also crucial for gene expression (Tosar and Cayota, 2020). During translation, the tRNAs must interact with the A, P, and E sites of the ribosomes (Edwards et al., 2020). Correct binding and movement of the tRNAs are essential for peptide chain elongation and protein synthesis. The ribosomes accurately identify and select those tRNAs that match the mRNA codons through complementary pairing between the tRNA anticodons and mRNA codons (Zhu et al., 2020). The accuracy of this process is vital for ensuring faithful protein synthesis, and any factors affecting this process can lead to abnormal gene expression (Demeshkina et al., 2010).

## 3 Exosome biogenesis and its role in intercellular communication

Exosomes are nanoscale vesicles secreted by the cells that possess unique biogenesis, secretion, and uptake mechanisms underpinning their crucial roles in intercellular communications (Lai et al., 2022). The biogenesis of exosomes is a complex and tightly regulated intracellular process involving multiple steps and cellular components.

The process begins with endocytosis, where cells internalize extracellular materials or membrane-associated molecules to form early endosomes that are acidic membrane-bound vesicles responsible for initial sorting of the internalized cargo (Krylova and Feng, 2023). As these endosomes mature, they undergo a series of changes leading to the formation of multivesicular bodies (MVBs) that are characterized by the presence of multiple intraluminal vesicles (ILVs). The creation of ILVs is a key step in exosome biogenesis that involves the budding and invagination of the endosomal membrane through two primary mechanisms: endosomal sorting complexes required for transport (ESCRT)-dependent and ESCRT-independent pathways (Kimiz-Gebologlu et al., 2022).

In the ESCRT-dependent mechanism, the ESCRT complex comprising ESCRT-0, I, II, and III works to recognize and bind the ubiquitinated proteins on the membrane, thereby promoting membrane curvature and vesicle formation. Here, ESCRT-0 identifies the ubiquitinated proteins, while ESCRT-I and II facilitate membrane deformation, and ESCRT-III mediates vesicle scission and release (Juan and Fürthauer, 2018). Conversely, the ESCRT-independent mechanism involves lipid-metabolizing enzymes like nSMase and PLD2, which alter the lipid composition and physical properties of the membrane to promote invagination and vesicle formation (Bavafa et al., 2025).

Mature MVBs release ILVs as exosomes by fusing with the plasma membrane, and this process is regulated by the Ras-related GTP-binding proteins (Rab GTPases) and SNARE proteins (Yang et al., 2020). The Rab GTPases like Rab27A, Rab27B, and Rab35 control MVB transport and fusion, while the SNARE proteins like VAMP7 and syntaxin 4 drive fusion of the MVBs with the plasma membrane (Schäfer et al., 2012). Exosomes are defined by three core features, namely, a 30–150 nm lipid bilayer structure, characteristic molecular markers, and specific biogenesis pathways. Their molecular markers are critical for identification and purification validation and fall into three main categories as follows: 1) *tetraspanin family proteins (surface markers).* In addition to the well-known CD63, CD81, and CD9 (Colombo et al., 2014), exosomes also express CD53 and CD37 consistently; these proteins mediate exosome membrane fusion and intercellular adhesion, which are essential for exosomal ncRNA delivery to the target cells. 2) *Cytoplasmic proteins (intraluminal or membrane-associated markers).* Exosomes contain proteins involved in their biogenesis, such as TSG101 and Alix, which confirm the origin of the exosomes from the endosomal pathways (Colombo et al., 2014); additionally, heat-shock proteins are enriched in the exosomes, which facilitate ncRNA folding and protect them from degradation during transport. 3) *Lipid-associated markers*. Exosomal membranes are enriched in cholesterol, sphingomyelin, and phosphatidylserine; these lipids enhance membrane rigidity and mediate exosome uptake by the recipient cells via phosphatidylserine receptors (Kimiz-Gebologlu et al., 2022). After being released as exosomes, these ILVs can be taken up by the recipient cells through various mechanisms dependent on the markers, allowing their ncRNA cargo to influence the recipient cell behaviors (Puno et al., 2019).

Understanding of exosome biogenesis is vital for elucidating their roles in intercellular communications as well as physiological and pathological processes. Exosomes are involved in normal physiological processes like immune regulation (Robbins and Morelli, 2014), tissue repair (Ha et al., 2020), and cellular signaling (Dixson et al., 2023), as well as in disease development, particularly in diabetes. In diabetes, the exosomes influence insulin resistance, β-cell dysfunction, immune modulation, and complications. For instance, exosomes from the muscle and fat cells contain miR-1 and miR-133, which target IRS-1 and INSR, inhibiting insulin signaling and leading to insulin resistance (Wang et al., 2022). Inflammatory environments caused by obesity can trigger macrophages to secrete proinflammatory exosomes that exacerbate insulin resistance (Rohm et al., 2024). Additionally, exosomes from diabetic patients show distinct miRNA and protein profiles compared to healthy individuals, highlighting their potential as biomarkers for the diagnosis and prognosis of diabetes (Lakey et al., 2023).

Exosomes also hold promise for diabetes therapy as drug delivery systems (especially for ncRNAs) and therapeutic targets. Their ability to specifically deliver the ncRNAs or other drugs to the target cells can enhance therapeutic efficacy. Overall, exosomes are pivotal in cellular communications and disease processes, thereby offering significant potential for the development of new diagnostic tools and therapeutic strategies in diabetes.

## 4 Roles of exosomal ncRNAs in DFUs

### 4.1 ncRNAs in DFUs

In recent years, the regulatory roles of ncRNAs in the pathogenesis of DFUs have attracted increasing attention, and related research findings have emerged as new potential therapeutic targets and strategies for DFUs.

miRNAs are key regulators of DFU development and their core therapeutic targets; their abnormal expression can directly drive the progression of DFUs, and their targeted modulation can achieve promising therapeutic effects. In terms of DFU development, under diabetic conditions, dysregulated miRNAs disrupt multiple wound-healing processes that are critical for preventing DFU progression. For example, miR-221-3p is upregulated in the keratinocytes under diabetic conditions, targeting DYRK1A and STAT3 phosphorylation to inhibit inflammation and promote skin wound healing in diabetic mice (Hu et al., 2024). Additionally, miR-217 is significantly upregulated in both DFU patients and rat models; by directly targeting HIF-1α, it inhibits the HIF-1α/VEGF pathway, leading to impaired angiogenesis that is a major factor contributing to the delayed healing of DFUs (Lin et al., 2019). Similarly, reactive oxygen species (ROS) accumulate in diabetic wounds and elevate miR-200c levels, suppressing the expression of anti-inflammatory factors and inhibiting endothelial cell migration, resulting in chronic tissue inflammation and endothelial dysfunction that further exacerbate DFU severity (D’Agostino et al., 2025). Conversely, miR-497 is downregulated in diabetic wounds, failing to inhibit the production of proinflammatory cytokines like IL-1β, IL-6, and TNF-α; this persistent inflammation hinders wound repair and promotes DFU formation (Ban et al., 2020). In terms of DFU treatment, targeting these dysregulated miRNAs has shown significant efficacy. For instance, topical administration of anti-miR-200c (a miRNA inhibitor) combined with catalase was shown to reduce ROS-induced miR-200c overexpression, restoring endothelial function and resolving chronic inflammation to significantly accelerate wound closure in diabetic mice (D’Agostino et al., 2025). Additionally, a graphene-oxide-based thermosensitive hydrogel has been developed to deliver exosomal miR-21 derived from ADSCs; this system modulates the PVT1/PTEN/IL-17 axis, suppressing excessive inflammation and promoting keratinocyte proliferation, which have been validated to enhance DFU wound healing in preclinical models (Chen et al., 2022).

LncRNAs mediate DFU development through competing endogenous RNA (ceRNA) mechanisms or protein-binding interactions, and their targeted regulation has been shown to provide novel therapeutic avenues for DFUs. In DFU development, dysregulated lncRNAs disrupt cell functions and tissue repair processes. For instance, the lncRNA URIDS is highly expressed in diabetic skin, where it binds to procollagen-lysine PLOD1 to inhibit collagen cross-linking; this disruption impairs extracellular matrix remodeling, which is a crucial step in wound healing, thereby delaying DFU recovery (Hu et al., 2020). Similarly, the lncRNA GAS5 is downregulated in the skin tissues of DFU patients. Its deficiency reduces binding to TAF15, leading to inactivation of the HIF-1α/VEGF pathway and subsequent angiogenesis defects, which are closely associated with the non-healing phenotype of DFUs (Peng et al., 2021). Additionally, research has shown that expression of the lncRNA NEAT1 is decreased in chronic DFU patients; this lncRNA acts as a sponge for miR-146a-5p. Silencing the lncRNA NEAT1 was shown to reduce the expression of angiogenic markers like mafG, SDF-1α, and VEGF, thereby regulating angiogenesis in DFUs (Architha et al., 2024). The lncRNA CASC2 was found to be downregulated in DFU patients and mice, with corresponding upregulation of miR-155. He et al. (2022) revealed that CASC2 directly targets miR-155 and that its overexpression promotes fibroblast proliferation and migration while inhibiting apoptosis through the miR-155/HIF-1α pathway. In DFU treatment, restoring or inhibiting specific lncRNAs can reverse the associated pathological processes. For instance, the exosomal lncRNA H19 derived from hypoxia-pretreated ADSCs acts as a sponge for miR-29b, upregulating FBN1 expression to enhance fibroblast proliferation and migration while inhibiting apoptosis; this exosomal delivery strategy was shown to significantly accelerate DFU wound healing in both *in vitro* and *in vivo* studies (Li et al., 2021a). Additionally, silencing the lncRNA SNHG16 (which is overexpressed in DFU tissues) relieves its sponging effect on miR-31-5p, restoring miR-31-5p-mediated promotion of keratinocyte migration and invasion to promote DFU wound repair (Chen et al., 2023a).

circRNAs have high stability in diabetic tissues and are therefore emerging as critical regulators of DFU development as well as promising therapeutic targets, primarily acting through miRNA sponging to modulate the DFU-related pathways. In DFU development, abnormal circRNA expression disrupts the wound-healing cascade. For example, circ_0080968 is upregulated in DFU wound tissues; it sponges miR-145-5p (a miRNA that promotes keratinocyte migration), leading to reduced miR-145-5p levels, suppressed keratinocyte migration, and impaired re-epithelialization that are key factors contributing to the chronicity of DFUs (Fu et al., 2023). Additionally, circ_0084443 is highly expressed in the wound tissues of DFU patients and promotes keratinocyte proliferation as well as migration by regulating the HBEGF/HIF-1α and PI3K, EGFR, and ERK signaling pathways to accelerate wound healing (Li et al., 2022). In contrast, circ_0001052 is downregulated in high-glucose-treated human umbilical vein endothelial cells (HUVECs); its deficiency fails to sponge miR-106a-5p, resulting in inhibited activation of the FGF4/p38MAPK pathway and reduced endothelial cell tube formation, which exacerbate angiogenesis defects in DFUs (Liang et al., 2022). In DFU treatment, circRNA-based therapeutic strategies have shown great potential. For example, a lipid-nanoparticle-delivered VEGF-A circRNA system was shown to achieve sustained VEGF-A expression in DFU wounds, directly promoting angiogenesis and accelerating wound healing in diabetic mice (Liu et al., 2024). These studies collectively reveal the complex regulatory network of circRNAs in DFU pathology. By interacting with miRNAs and mRNAs, the circRNAs modulate critical processes like cell proliferation, migration, apoptosis, and inflammatory responses to affect wound healing (Fu et al., 2023; Li et al., 2022; Liu et al., 2024). Although the specific mechanisms and targets vary across studies, all works offer new insights and potential therapeutic targets for DFU treatment. Future research should therefore delve deeper into the dynamic expression and functions of circRNAs at different stages of DFU development to derive more precise clinical strategies.

Other ncRNAs like the piRNAs, tRNAs, and snoRNAs also show potential in DFU treatment. PiR-8087 targets the PTEN/AKT1 pathway to promote cell proliferation and migration (Zhang et al., 2023c). tRF-Gly-CCC-039 is significantly upregulated in the DFU tissues and high-glucose models; it inhibits the proliferation, migration, and tube formation of HUVECs as well as downregulates the expression of repair-related molecules, thereby impairing vascular angiogenesis and tissue repair while delaying DFU healing (Zhang et al., 2023d). The snoRNA U87 regulates VEGF expression to promote angiogenesis (Makarova and Kramerov, 2005).

**FIGURE 2:**
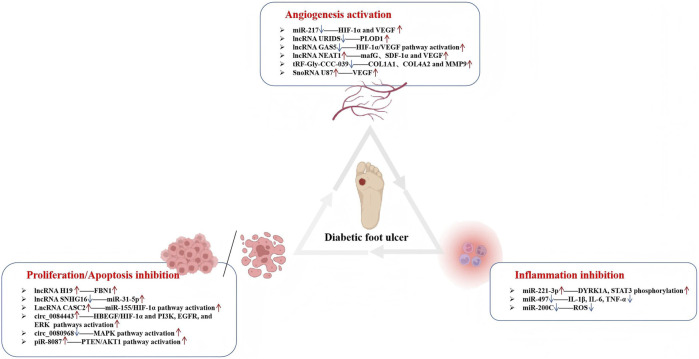
Regulatory networks of exosomal non-coding RNAs in diabetic foot-ulcer (DFU) healing. The diagram summarises pro-healing mechanisms delivered by exosomal ncRNAs: angiogenesis activation (top), proliferation/apoptosis inhibition (left) and inflammation inhibition (right). Up- or down-regulation of specific miRNAs, lncRNAs, circRNAs, piRNAs, tRFs and snoRNAs modulates key signalling axes (HIF-1α/VEGF, PTEN/AKT, MAPK, etc.) to accelerate re-epithelialisation, granulation-tissue formation and wound closure in DFU.

In conclusion, ncRNAs have diverse roles in the pathogenesis of DFUs as they affect key processes like inflammation, angiogenesis, cell proliferation, and cell apoptosis through their abnormal expression (see Figure 2). Further research into the regulatory mechanisms of ncRNAs could provide new therapeutic strategies and targets for DFU.

### 4.2 Applications and prospects of exosomes in the treatment of DFUs

Exosomes show great potential in the treatment of DFUs (Zhu et al., 2025). Recent studies have shown their roles in promoting wound healing, antibacterial actions, and inflammation regulation (Wang et al., 2025a). DFU is a serious complication of diabetes that is caused by microvascular disease, nerve damage, immune dysfunction, and metabolic disorders; it makes wound healing difficult, which can cause pain and increase the medical burden on the patient (Ranuve and Mohammadnezhad, 2022). In this context, exosomes are gaining attention as a new therapy against DFUs.

Exosomes can regulate cell functions and promote ulcer healing as a new strategy for DFU treatment. With advantages like low immunogenicity, high stability, and high modifiability, exosomes serve as ideal drug delivery vehicles (Raghav et al., 2021). They can deliver therapeutic molecules like miRNAs and proteins to modulate the pathogenesis of DFU (Wu et al., 2024). Mesenchymal stem cell exosomes (MSC-Exos) have repair functions similar to MSCs but without the risk of tumorigenesis and are easier to apply (Zhou et al., 2023). They transfer bioactive molecules like lipids, proteins, and RNA to regulate the target cell behaviors for wound healing. For example, in high-glucose conditions, adipose-derived MSC-Exos can activate the AMPK pathway or restore IRS-1 and PKB phosphorylation to reduce oxidative stress, improve insulin resistance, relieve β-cell apoptosis, and effectively treat diabetic wounds and ulcers (Guo et al., 2024). Moreover, exosomes derived from pioglitazone-pretreated MSCs promote endothelial cell angiogenesis via the PI3K/AKT/eNOS pathway to accelerate diabetic wound healing (Hu et al., 2021). In bacterial-infection-related DFU treatment, MSC-Exos inhibit M1 macrophage polarization, promote M2 polarization, lower the inflammatory factors, and are rich in growth factors and therapeutic ncRNAs, offering antibacterial and regenerative effects (Qu et al., 2022). Additionally, Li et al. (2018) assessed the impacts of exosomes from ADSCs on high-glucose-induced endothelial progenitor cell (EPC) senescence and explored the enhancing effect of Nrf2 overexpression on ADSC exosome function. The results showed that ADSC exosomes alleviate high-glucose-induced EPC senescence and promote angiogenesis. Nrf2 overexpression further enhances their protective effects by reducing ROS and inflammatory factor levels in EPCs, improving cell viability and tube formation ability.

Exosome functionalization is critical for efficient loading of therapeutic ncRNAs and maintenance of exosome bioactivity and is achieved through five mainstream methods. These methods have distinct efficacy profiles that determine their suitability for DFU treatment (Table 1): 1) *Sonication.* This method uses low-frequency ultrasound to create transient pores in the exosomal membrane that enable ncRNA entry. Its key advantages include simple operation procedures, low equipment costs, and minimal disruption to the exosome lipid bilayer integrity (loading efficiency: approximately 30–50% for miRNAs) (Yang et al., 2020). However, high-intensity sonication can cause partial exosome rupture (approximately 10–15% loss) and ncRNA degradation, limiting its application in large-scale production (Bavafa et al., 2025). For DFU, sonication is an ideal approach for loading small ncRNAs (e.g., miR-21 and miR-31-5p) owing to its low toxicity, where its compatibility with hydrogel encapsulation (Wang et al., 2025b) helps offset any stability limitations. 2) *Extrusion.* This method forces the exosomes and ncRNAs to pass through a porous membrane (50–100 nm pores) to induce membrane fusion between them and cargo loading. It ensures high uniformity in the size of the exosomes after functionalization and a higher loading efficiency (45–60% for lncRNAs) compared to sonication (Kimiz-Gebologlu et al., 2022). The main drawbacks of this approach are low throughput and potential membrane damage under high pressure (5–8% exosome aggregation). In DFU research, extrusion is preferred for loading large ncRNAs owing to its ability to preserve the ncRNA structural integrity, although it requires subsequent integration with microneedle patches to improve wound-healing retention (Senneville et al., 2024). 3) *Freeze–thaw cycles.* Repeated cycles of freezing (−80 °C) and thawing (room temperature) temporarily disrupt the exosomal membranes, allowing ncRNA entry. This method is cost-effective and requires no specialized equipment but offers moderate loading efficiency (25–40% for circRNAs) (Lai et al., 2022). However, it often leads to exosome aggregation (20–25%) and reduced *in vivo* stability, which can be mitigated by combining the freeze-thawed exosomes with self-healing hydrogels (Wang et al., 2019). For DFU, the freeze–thaw method is suitable in preclinical studies or low-resource settings but requires stability enhancement for clinical use. 4) *Electroporation.* An electric field is applied in this method to create transient membrane pores and achieve the highest loading efficiency (60–80% for most ncRNAs) among the five methods (Kimiz-Gebologlu et al., 2022). It is particularly effective for nucleic acid cargoes and preserves the exosome surface markers (e.g., CD63, CD81) that are critical for target cell uptake. The primary limitation of this method is exosome damage induced by high voltage (15–20% loss), which can be addressed by optimizing the voltage parameters (100–200 V/cm for ncRNA loading). Electroporation is the most widely used method in preclinical studies on DFUs (Zhu et al., 2025) as its high efficiency ensures sufficient ncRNA delivery to the hypoxic wound tissues. 5) *Chemical transfection.* Here, cationic reagents (e.g., liposomes and polyethyleneimine) mediate ncRNA binding to the exosome surfaces and subsequent internalization. It offers high loading efficiency (55–70%) and ease of scalability (Yang et al., 2020). However, the residual cationic reagents may exhibit cytotoxicity (resulting in a 10–15% reduction in fibroblast viability) and can trigger *in vivo* immune clearance, limiting application to DFU wounds where the tissue repair capacity is fragile (Qu et al., 2022). Chemical transfection is rarely used for DFUs but may have potential if combined with biocompatible reagents.

**TABLE 1:** Efficacy comparisons of exosome functionalization methods for the treatment of DFUs.

Functionalization method	Loading efficiency (ncRNA)	Exosome integrity (%)	*In* *vivo* stability	DFU applicability	Key limitation for DFU	References
Sonication	30%–50% (miRNAs)	85–90	Moderate	High (small ncRNAs)	Partial ncRNA degradation	Bavafa et al. (2025); Yang et al. (2020)
Extrusion	45%–60% (lncRNAs)	92–95	High	High (large ncRNAs)	Low throughput	Senneville et al. (2024); Kimiz-Gebologlu et al. (2022)
Freeze–thaw cycles	25%–40% (circRNAs)	75–80	Low	Moderate (with hydrogels)	Aggregation	Lai et al. (2022); Wang et al. (2019)
Electroporation	60%–80% (all ncRNAs)	80–85	High	Highest	Voltage-induced damage	Kimiz-Gebologlu et al. (2022); Zhu et al. (2025)
Chemical transfection	55%–70% (all ncRNAs)	88–92	Moderate	Low	Cytotoxicity	Qu et al. (2022)

As a core delivery vehicle in DFU treatment, exosomes exhibit distinct differences in perspectives regarding the intracellular delivery mechanisms and functional effects of drugs/proteins *versus* ncRNAs.

From the perspective of drug/protein delivery, exosomes that rely on their low-immunogenicity lipid bilayer structure and membrane fusion capability can directly deliver the growth factors, antibacterial proteins, or small-molecule drugs into the target cells (Zhao et al., 2024). These molecules do not require complex gene regulatory pathways after entering the cells and can quickly exert their biological effects; for instance, VEGF carried by exosomes can directly bind to the VEGF receptors on the surfaces of the vascular endothelial cells, thereby transiently activating the PI3K/AKT/eNOS pathway to promote angiogenesis (Li et al., 2021b), while exosome-encapsulated antibacterial peptides can directly disrupt the cell membranes of pathogenic bacteria (e.g., *Staphylococcus aureus*) in DFU wounds (Mukerjee et al., 2024) to rapidly control the spread of infection.

In contrast, the exosomal delivery of ncRNAs relies more on the “indirect regulatory perspective” of post-transcriptional regulation: exosomes protect the ncRNAs from degradation by extracellular nucleases and enable precise targeted delivery (Zhang et al., 2015). Once inside the cells, miRNAs can bind to the 3′-UTR of the target mRNAs to inhibit or promote their translation (Yue et al., 2020); lncRNAs can reshape gene expression networks by sponging miRNAs or binding to transcription factors (Yue et al., 2020); circRNAs can leverage the high stability of their circular structures to achieve long-term regulation by sponging miRNAs or interacting with proteins (Kok and Yu, 2020). Although this mode lacks the direct effects of proteins or drugs, it can achieve sustained wound repair by adjusting the intracellular signaling pathways.

These differences in the delivery perspectives further extend to the functional impacts on different cell types involved in the DFU process. For keratinocytes, EGF delivered by the exosomes can directly stimulate cell activation to accelerate re-epithelialization, while miR-21 carried by the exosomes inhibits inflammation and promotes cell proliferation and migration via the PVT1/PTEN/IL-17 axis, thereby achieving a repair effect described as “rapid initiation with long-term maintenance” (Chen et al., 2022). For fibroblasts, the TGF-β protein in exosomes can directly induce differentiation into myofibroblasts to enhance wound contraction, while the lncRNA H19 promotes ordered synthesis of collagen by upregulating FBN1, thus reducing scar formation (Li et al., 2021a). In HUVECs, the VEGF protein delivered by exosomes can quickly activate cell proliferation signals while circHIPK3 continuously promotes lumen formation via the miR-20b-5p/Nrf2/VEGF-A axis, with the two working synergistically to improve wound ischemia (Liang et al., 2023). For macrophages, the IL-10 protein carried by exosomes can directly inhibit the activation of M1-type macrophages to reduce inflammation, while miR-155 inhibitors (delivered via exosomes) can downregulate proinflammatory factors to drive the polarization of macrophages toward the M2 type to promote tissue repair (Ye et al., 2017). Additionally, exosomes can regulate the function of ADSCs; for example, exosomes carrying Nrf2 can enhance the antioxidant capacities of the ADSCs, enabling them to secrete more reparative cytokines and thus indirectly participate in wound healing (Chen et al., 2023b). These delivery mechanisms and cellular effects from different perspectives complement each other, collectively forming a multitarget multistage action system of exosomes in DFU treatment.

Combining functionalized exosomes with biomaterials (e.g., hydrogels, microneedles) can further enhance the therapeutic efficacy: for example, electroporated exosomes loaded with miR-21 and encapsulated in methacrylated gelatin hydrogels exhibit a 2.3-fold higher wound closure rate in diabetic mice than unfunctionalized exosomes (Zhu et al., 2025). Additionally, a microneedle patch developed using methacrylated gelatin and polyethylene glycol diacrylate was designed to deliver HUVEC-derived exosomes and tazarotene to promote diabetic wound healing; this microneedle patch exhibits good biocompatibility and controllable drug-release properties, effectively promoting cell migration, angiogenesis, and wound healing. It enables targeted delivery of the exosomes, thereby improving their bioavailability and enhancing the repair of diabetic wounds (Yuan et al., 2022).

In conclusion, exosomes provide a promising therapeutic strategy for the treatment of DFUs with broad application prospects. Future research efforts should focus on optimizing the exosome preparation and delivery methods, further exploring their action mechanisms, and conducting more clinical trials to advance the clinical applications of exosomes to treat DFUs.

## 5 Roles of exosomal ncRNAs in DFUs

In recent years, there have been in-depth research efforts on the pathogenesis of diabetic foot, and exosomes have emerged as key players in cellular communications. These lipid nanovesicles secreted by the cells carry various bioactive molecules, including ncRNAs, and hold significant promise for the treatment of DFUs (see Table 2).

**TABLE 2:** Roles of exosomal ncRNAs in DFUs.

ncRNA	Exosome source	Target gene	Mechanism	Reference
miR-21	ADSCs	*PVT1*	Competitively binds to PVT1, preventing it from modulating the activity of the PI3K/Akt signaling pathway, which in turn curtails the proliferation of HUVECs	Chen et al. (2022)
miR-195-5p and miR-205-5p	Wound fluids of DFUs	*VEGF-A*	Inhibiting the expression of VEGF-A directly to the 3′-UTR of VEGF-A and in turn promoting an inhibitory effect of DF-EVs on angiogenesis	Liu et al. (2021)
miR-26b-5p	Platelet-rich plasma	*MMP-8*	Disrupting neutrophil extracellular trap formation in diabetic wounds by silencing MMP-8	Rui et al. (2024)
miR-15a-3p	Diabetic patients’ blood	*NOX5*	Suppressing the NOX5/ROS signaling pathway and delaying wound healing	Xiong et al. (2020)
miR-155	HUVECs	Not mentioned	Reducing inflammation, enhancing angiogenesis and collagen formation, and accelerating healing	Ye et al. (2017)
miR-31-5p	Milk	*HIF1AN*	Improving cellular uptake and stability and promoting endothelial cell functions and angiogenesis	Yan et al. (2022)
lncRNA H19	Hypoxia-treated ADSCs	Not mentioned	Improved cell proliferation, migration, and angiogenesis in H_2_O_2_-triggered HUVECs	Qian et al. (2024)
lncRNA H19	ADSCs	*miR-130b-3p*	Promoting macrophage M2 polarization, thereby enhancing fibroblast proliferation, migration, and endothelial cell angiogenesis	Li et al. (2023)
lncRNA H19	HF-MSC	Not mentioned	Inhibiting NLRP3 pyroptosis to promote diabetic mouse skin wound healing	Yang et al. (2023)
lncRNA MALAT1	Human keratinocyte	*miR-1914-3p*	Suppressing miR-1914-3p to activate MFGE8 and eventually promote wound healing by enhancing macrophage phagocytosis, converting to a pro-healing phenotype and reducing apoptosis	Kuang et al. (2023)
lncRNA KLF3-AS1	BMSCs	*miR-383*	Downregulating miR-383 and boosting the expression of VEGF-A	Han et al. (2022)
LINC01435	High-glucose-pretreated HaCaT	*YY1*	Decreasing tube formation and ability of HUVECs to migrate as well as inhibiting angiogenesis	Fu et al. (2022)
lncRNA SENCR	Human ADSCs	*DKC1*	Facilitating the wound-healing process by increasing angiogenesis	Sun et al. (2022)
circ-Snhg11	BMSCs	*miR-144-3p*	Enhancing angiogenesis along with reduction in GPX4-mediated ferroptosis	Tang et al. (2024a)
circ-0001747	ADSCs	*miR-199a-5p*	Sponging miR-199a-5p and upregulating HIF-1α to promote healing	Wang et al. (2025c)
circ-0001052	ADSCs	*miR-106a-5p*	Activating the p38/MAPK pathway to improve cell damage	Liang et al. (2022)
circ-Astn1	ADSCs	*miR-138-5p*	Upregulating SIRT1, promoting angiogenesis, and inhibiting apoptosis	Wang et al. (2023c)
circ-Erbb2ip	ADSCs	*miR-670-5p*	Promoting wound healing by targeting the miR-670-5p/Nrf1 pathway	Tang et al. (2024b)
circHIPK3	HUVECs	*miR-20b-5p*	Promoting angiogenesis via the miR-20b-5p/Nrf2/VEGF-A axis	Liang et al. (2023)
circ-ITCH	BMSCs	*TAF15*	Activating of the Nrf2 signaling pathway by recruiting TAF15 protein to accelerate wound healing	Chen et al. (2023b)

ADSCs, adipose-derived mesenchymal stem cells; PVT1, plasmacytoma variant translocation 1; PI3K/Akt signaling pathway, phosphoinositide 3-kinase/Akt signaling pathway; HUVECs, human umbilical vein endothelial cells; DFUs, diabetic foot ulcers; VEGF-A, vascular endothelial growth factor A; 3′-UTR, 3′-untranslated region; DF-EVs; exosomes isolated from diabetic foot ulcer wound fluid; MMP-8, matrix metalloproteinase-8; NOX5, NADPH oxidase 5; ROS, reactive oxygen species; HIF1AN, hypoxia-inducible factor 1 subunit alpha inhibitor; HF-MSC, hair follicle mesenchymal stem cells; NLRP3, NLR family pyrin domain-containing 3; MFGE8, milk fat globule-EGF factor 8; BMSCs, bone marrow mesenchymal stromal cells; YY1, Yin Yang 1; DKC1, dyskerin pseudouridine synthase 1; GPX4, glutathione peroxidase 4; HIF-1α, hypoxia-inducible factor 1α; p38/MAPK pathway, p38/mitogen-activated protein kinase pathway; SIRT1, Sirtuin 1; Nrf1, nuclear respiratory factor 1; Nrf2, nuclear factor erythroid 2-related factor 2; TAF15, TATA-binding protein-associated factor 15.

Exosomal miRNAs have shown significant promise in diabetic foot therapy. For instance, a thermosensitive hydrogel based on graphene oxide was developed to deliver miR-21 to promote wound healing in diabetic mice. This hydrogel boosts miR-21 levels in ADSC-derived exosomes, modulating the PVT1/PTEN/IL-17 axis to facilitate wound healing. Bioinformatics and experiments have identified PVT1 as a crucial lncRNA that can bind with miR-21 to influence the PI3K/Akt pathway and promote healing (Chen et al., 2022). In contrast, exosomes isolated from DFU wound fluid (DF-EVs) can suppress the proliferation, migration, and angiogenesis of HUVECs to delay healing. Further analysis revealed that miR-195-5p and miR-205-5p are upregulated in DF-EVs, directly targeting the 3′-UTR of VEGF-A to inhibit its expression while affecting angiogenesis and healing (Liu et al., 2021). Platelet-rich plasma-derived exosomes (PRP-Exos) also show wound-healing potential in diabetic environments. Their miR-26b-5p targets MMP-8 to inhibit neutrophil extracellular traps (NETosis) and promote healing (Rui et al., 2024). Conversely, circulating exosomal miR-15a-3p in diabetic patients’ blood exosomes (Dia-Exos) can impede wound repair; miR-15a-3p is upregulated in Dia-Exos and targets the NOX5/ROS pathway to suppress endothelial cell function and delay healing (Xiong et al., 2020). Additionally, miR-155 inhibition accelerates healing in diabetic rats, reducing inflammation in the wounds while enhancing angiogenesis and collagen formation (Ye et al., 2017). A cow-milk-exosome-based miR-31-5p delivery system also showed promise in improving cellular uptake and stability while promoting endothelial cell functions and angiogenesis in experiments (Yan et al., 2022).

Notably, exosomes derived from lipid-dysregulated adipocytes carry miR-1 and miR-133 that target IRS-1 and INSR, thereby exacerbating insulin resistance (Wang et al., 2022). This creates a feedforward loop where impaired lipogenesis/lipid accumulation promotes exosomal ncRNA release, further worsening metabolic dysfunction and increasing DFU susceptibility.

Exosomal lncRNAs are also important in diabetic foot treatment. For example, numerous studies have confirmed that the exosomal lncRNA H19 from various sources is effective in treating DFUs. The lncRNA H19 from hypoxia-treated ADSC-derived exosomes can promote cutaneous wound healing by enhancing cell proliferation, migration, and angiogenesis *in vitro* and *in vivo*. The mechanism involves the USP22/HIF-1α/H19 axis, where hypoxia-treated ADSCs carrying USP22 stabilize HIF-1α, which transcriptionally activates H19 (Qian et al., 2024). Li et al. (2023) showed that the lncRNA H19 carried by exosomes from ADSCs can promote the polarization of macrophages to the anti-inflammatory M2 phenotype, thereby accelerating skin wound healing. They further demonstrated that H19 promotes wound healing by binding to miR-130b-3p and modulating the expression of PPARγ and STAT3. The lncRNA H19 in hair follicle mesenchymal stem cell (HF-MSC)-derived exosomes can also significantly accelerate wound healing, reduce inflammatory cells, and lower caspase-1, IL-1β, and TNF-α levels to inhibit the NLRP3 inflammatory response (Yang et al., 2023). The lncRNA MALAT1 in keratinocyte-derived exosomes promotes healing by upregulating MFGE8—an effect achieved by competing with miR-1914-3p for binding—to affect macrophage function and the TGFB1/SMAD3 pathway (Kuang et al., 2023). Bone marrow mesenchymal stromal cell (BMSC)-derived exosomal lncRNA KLF3-AS1 stimulates angiogenesis via miR-383, thereby activating the VEGF-A axis (Han et al., 2022). However, LINC01435 in high-glucose HaCaT cell-derived exosomes alters YY1 localization in HUVECs and upregulates HDAC8, in turn inhibiting endothelial cell migration and tube formation to ultimately impact angiogenesis (Fu et al., 2022). Sun et al. (2022) found that hypoxia-treated ADSC-derived exosomes can significantly promote the proliferation, migration, and angiogenesis of HUVECs. Studies have shown that the lncRNA SENCR in hypoxia-treated-ADSC-Exos interacts with EGR-1 to activate the VEGF-A axis, thus promoting angiogenesis. Specifically, EGR-1 binds to the promoter region of lncRNA SENCR, upregulating its expression. Then, SENCR interacts with DKC1 to maintain stable expression of VEGF-A.

Exosomal circRNAs play crucial roles in diabetic foot treatment. For example, BMSC-derived exosomes deliver circ-Snhg11, which sponges miR-144-3p to enhance ferroptosis resistance via the SLC7A11/GPX4 pathway to accelerate wound healing (Tang et al., 2024a). Wang et al. (2025c) found that circ-0001747 was more highly expressed in hypoxia-preconditioned ADSC-derived exosomes than ADSC-derived exosomes treated under normoxic conditions. Moreover, overexpression of circ-0001747 significantly accelerated wound healing in DFU mice. Mechanistically, circ-0001747 acted as a sponge for miR-199a-5p, thereby increasing HIF-1α expression and subsequently promoting angiogenesis, in addition to inhibiting cell apoptosis and ROS generation. Circ-0001052 in high-glucose HUVECs is downregulated, but its overexpression improves cell damage by sponging miR-106a-5p and upregulating FGF4, thereby activating the p38/MAPK pathway (Liang et al., 2022). Circ-Astn1 promotes angiogenesis and inhibits apoptosis by adsorbing miR-138-5p and upregulating SIRT1 while downregulating FOXO1 (Wang et al., 2023c). Circ-Erbb2ip in hypoxia-preconditioned ADSC-secreted exosomes regulates the miR-670-5p/Nrf1 axis (Tang et al., 2024b). CircHIPK3 in umbilical-cord-MSC-derived exosomes promotes angiogenesis via the miR-20b-5p/Nrf2/VEGF-A axis (Liang et al., 2023). Additionally, small extracellular vesicles released during bone transport, which are rich in antioxidants and miRNAs (notably miR-494-3p), promote healing particularly when combined with the ginsenoside Rg1. Circ-ITCH in BMSC-derived exosomes enhances healing by activating Nrf2 and inhibiting ferroptosis (Chen et al., 2023b).

One of the key challenges in utilizing exosomal ncRNAs for DFU treatment is the development of an effective delivery method (Zhang et al., 2023e). Exosomes are natural carriers of ncRNAs and offer several advantages, including low immunogenicity, high stability, and modifiability (Zhang et al., 2023f). However, ensuring efficient delivery of exosomes to the wound site while avoiding immune clearance and degradation is crucial (Ye et al., 2024). Several strategies, including sonication, extrusion, freeze–thaw cycles, electroporation, and chemical transfection, have been proposed to enhance exosome delivery; these methods improve the therapeutic efficacies of the exosomes by boosting their stability and targeting capabilities (Sharma et al., 2025).

Combining exosomes with biomaterials like hydrogels and microneedle patches can prolong exosome retention at the wound site and enable sustained release, thereby enhancing their therapeutic efficacy further (Rahman et al., 2025). For example, Wang et al. (2019) developed an injectable self-healing antibacterial-peptide-based F127/OHA-EPL hydrogel for delivering ADSC-derived exosomes to promote chronic diabetic wound healing. This hydrogel has rapid self-healing ability, shear-thinning properties, and long-term pH-responsive exosome release behavior, which effectively promote the proliferation, migration, and tube formation of HUVECs to accelerate the healing of diabetic full-thickness skin wounds, increase wound closure rates, and promote granular tissue formation and collagen deposition.

In summary, while the potential of exosomal ncRNAs in DFU treatment is promising, significant challenges still remain with regard to enhancing the delivery methods and conducting clinical trials to validate their safety and efficacy. Future research efforts should thus focus on these areas to ensure the widespread clinical use of exosomal ncRNA-based therapies in DFU treatment.

## 6 Conclusion

Exosomal ncRNAs hold significant promise in the treatment of DFUs by enhancing wound healing through diverse mechanisms. However, several challenges must be addressed to maximize their therapeutic potential. Currently, the exosome extraction and purification methods used often lack consistency in yield and purity, where contaminants like protein aggregates or other extracellular vesicles could obscure the experimental results. Validating exosome identity using a panel of characteristic markers is thus critical: for example, Western blotting to detect TSG101/Alix (cytoplasmic markers) and CD63/CD81 (surface markers), when combined with flow cytometry to quantify CD9-positive vesicles, helps ensure the purity of exosomal ncRNA preparations (Lai et al., 2022; Colombo et al., 2014). The natural therapeutic capacities of exosomes may be insufficient for severe DFU cases, necessitating functional enhancement through techniques like sonication (for ncRNA loading) or chemical modification (for targeting optimization). Additionally, the design of efficient drug delivery systems remains crucial as exosomes must be effectively loaded with the therapeutic ncRNAs and optimally targeted to the wound site while avoiding immune clearance. Ensuring the stability of the exosomes and their ncRNA cargo *in vivo* is also essential as both can be degraded by nucleases or cleared by the immune system, which reduces the therapeutic effectiveness directly.

In the future, the development of exosomal ncRNA therapies for DFUs will likely focus on several key areas. Advances in exosome isolation techniques, such as affinity-based methods and microfluidic systems, will improve the preparation quality and consistency. Researchers may also continue to explore ways to engineer exosomes for enhanced targeting, stability, and drug-loading capacity; in this regard, three targeted delivery strategies show promise specifically for DFUs: 1) active targeting modification, where the exosome surfaces can be functionalized with ligands that bind to the receptors highly expressed in the DFU wound microenvironment; for example, RGD peptide targets integrin αvβ3 on the vascular endothelial cells in ischemic DFU tissues, while anti-CD44 antibodies target activated fibroblasts in the wound granulation tissue; this design guides the exosomes to accumulate at the ulcer site and reduce off-target distribution. 2) Biomaterial-based localized delivery, which integrate exosomes with DFU-specific biomaterials to enhance site retention; for instance, microneedle patches made of methacrylated gelatin and polyethylene glycol diacrylate can penetrate the hyperkeratotic layer of a DFU wound—a major barrier to topical delivery—and release exosomes in a sustained manner. Injectable thermosensitive hydrogels, such as F127/OHA-EPL, respond to a wound temperature of 37 °C to form a gel *in situ*, thereby trapping exosomes at the ulcer site for up to 72 h. 3) Physical auxiliary delivery, where low-intensity focused ultrasound can be used to temporarily increase the permeability of the wound tissue membrane and promote exosome internalization by the target cells, such as keratinocytes and endothelial cells. This approach has been validated in preclinical studies to improve exosomal ncRNA accumulation in the DFU wounds by ∼2.1-fold compared to passive delivery.

The integration of exosomes with biomaterials like hydrogels and microneedle patches will likely become a key strategy for sustained release. Increased focus on clinical trials will also be necessary to validate the safety and effectiveness of these therapies, bridging the gap between preclinical research and clinical application. Although the potential of exosomal ncRNAs in DFU treatment is significant, the translation of these therapies into clinical practice requires further validation; currently, there are no ongoing clinical trials involving exosomal ncRNAs for the treatment of DFUs. This area remains largely unexplored, highlighting the need for pioneering research to explore the therapeutic potential of exosomal ncRNAs in DFUs.

In summary, while the potential of exosomal ncRNAs in DFU treatment is promising, significant challenges remain in terms of optimizing exosome preparation, enhancing delivery methods, and conducting clinical trials to validate their safety and efficacy. Future research efforts should focus on these areas to ensure the widespread clinical use of exosomal ncRNA-based therapies in DFU treatment.
